# Hematoma-inspired injectable composite hydrogels incorporating hybrid metal ion microspheres for accelerated bone regeneration

**DOI:** 10.1016/j.mtbio.2025.102342

**Published:** 2025-09-25

**Authors:** Fan Cao, Jin-Yong Wu, Qing-Ning Wang, Jun-Jie Xiao, Zhu Chen, Ya-Wen Wang, Zhi-Guo Wang, Jia-Zhuang Xu, Zhong-Ming Li, Bai-Song Zhao

**Affiliations:** a Department of Cardiovascular Surgery, Guangdong Cardiovascular Institute, Guangdong Provincial People's Hospital, Guangdong Academy of Medical Sciences, Southern Medical University, Guangzhou, 510080, China; b College of Polymer Science and Engineering, State Key Laboratory of Advanced Polymer Materials, Sichuan University, Chengdu, 610065, China; c Department of Anesthesiology, The Affiliated Panyu Central Hospital of Guangzhou Medical University, Guangzhou, 511400, China; d Medicine and Engineering Interdisciplinary Research Laboratory of Nursing & Materials, West China Hospital, Sichuan University/West China School of Nursing, Sichuan University, Chengdu, 610041, China; e West China School of Medicine/West China Hospital, Sichuan University, Chengdu, 610041, China; f Department of Anesthesiology, Zhujiang Hospital, Southern Medical University, Guangzhou, 510280, China

**Keywords:** Bone defects, Injectable hydrogel, Osteogenic microspheres, Synergistic effect, Bone regeneration

## Abstract

Injectable hydrogels emerge as a particularly attractive candidate in bone tissue engineering, due to their minimally invasive administration and conformal filling for irregular bone defects. However, they still lack sufficient osteoinductive activities for effective bone repair. Herein, we engineer a hematoma-inspired injectable composite hydrogel consisting of polylactide microspheres encapsulated with magnesium oxide and strontium oxide and the alginate hydrogel crosslinked by slow release of Ca^2+^ for promoting bone regeneration in critical-sized bone defects. A complementary release of rapidly released Sr^2+^ and gradually released Mg^2+^ effectively promotes the proliferation, migration and osteogenic differentiation of bone marrow mesenchymal stem cells as well as biomineralization, mimicking the release of various growth factors from hematoma for expediting bone healing. The ionic crosslinking alginate composite hydrogel imitates the fibrin network of hematoma to serve as a temporary scaffold to support bone healing. The excellent injectability, high mechanical strength and good biocompatibility are also gathered. Through a critical-sized bone defect rat model, our hematoma-inspired hydrogel displays strong osteogenic ability to upregulate the expression of osteogenesis-related proteins and accelerate new bone formation, ascribed to the synergistic effect of released Mg^2+^ and Sr^2+^. Overall, the novel composite hydrogel offers a significant application potential for the treatment of bone defects.

## Graphical abstract

**Figure:**
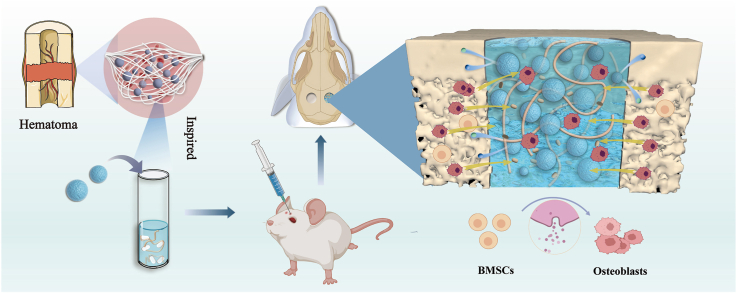

## 1 Introduction

Critical-sized bone defects arising from tumors, trauma, infections or congenital conditions are trapped in the dilemma of lacking the capacity to heal spontaneously [1,2]. While bone grafting is a standard approach [3], its limitations-such as donor site morbidity and immune rejection-highlight the urgent need for alternative bioactive materials [4,5]. Critical-sized bone defects entail substantial economic expenditures in healthcare and severe distress on patients and face a challenge in current clinical intervention [[6], [7], [8]]. Bone tissue engineering aims to construct a bone repair material system *in vitro* similar with the structure and properties of the bone tissue *in vivo* [9], and exhibits a promising potential in repairing bone defects. Among diverse substitutes for bone regeneration, hydrogels have gained significant attention as attractive bioactive materials for bone defects, owing to the manipulability, fluidityand biocompatibility [[10], [11], [12]]. The injectable feature enables hydrogels to accommodate the complex morphology of bone defects in a minimally invasive manner [13], offering mechanical support that integrates seamlessly with host tissues [14,15].

Considerable efforts have been devoted to fabricating injectable osteogenic hydrogels by encapsulating and continuously releasing therapeutic agents to construct a favorable microenvironment for cell proliferation and differentiation as well as tissue formation. Zhou et al. incorporated bone morphogenetic protein-2 (BMP-2) into the injectable hydrogel, confirming that the sustained-release BMP-2 from the hydrogel was conductive to promoting new bone formation [16]. Wang et al. prepared an injectable hydrogel loaded with dental pulp stem cell-derived extracellular vesicles to promote the expression of BMP-2 and alkaline phosphatase for accelerating bone tissue regeneration [17]. Nevertheless, obvious deficiencies of bioactive proteins including the poor stability, short shelf time and the high price severely restrict their wide applications [18].

Therapeutic metallic ions allure great attention in bone tissue engineering because of their osteogenic activity, stability and low cost. Liao et al. verified that the rapid release of magnesium ion (Mg^2+^) from the oxidized dextran-based injectable hydrogel was effective in treating the bone defect [19]. Wang et al. designed a gelatin methacryloyl (GelMA) hydrogel with releasing cerium ion to enhance the expression of osteogenic markers and promote bone regeneration [20]. The initial burst release of bioactive metallic ions fails to match the long-term repair process of bone defects and compromises the osteogenic effect. Recently, the cooperative action of different metallic ions has been designed to provide an appropriate ionic microenvironment for bone regeneration. Chen et al. prepared a chitosan oligosaccharide-based hydrogel with synchronous release of Mg^2+^ and zinc ions (Zn^2+^) [21]. On the basis of the osteogenic property of Mg^2+^, Zn^2+^ suppressed the osteoclast-mediated resorption. The synergistic osteogenic effect of Mg^2+^ and Zn^2+^ endowed the hydrogel with a good new bone formation capacity. Lyu et al. designed a double-layer hydrogel consisting of the upper GelMA/sodium alginate layer with Mg^2+^ and the bottom GelMA/poly(ethylene glycol) diacrylate layer with calcium ion (Ca^2+^) [22]. The early release of Mg^2+^ facilitated the nerve regeneration and osteogenic-related neuropeptide release, and the sustained release of Ca^2+^ supported the long-term osteogenesis. The double-layer hydrogel presented the accelerated nerve network reconstruction and bone regeneration. Although large progress has been made, it is stilled challenged and highly desired to develop injectable hydrogels with continuous delivery of metallic ions for effective bone regeneration.

**Fig. 1:**
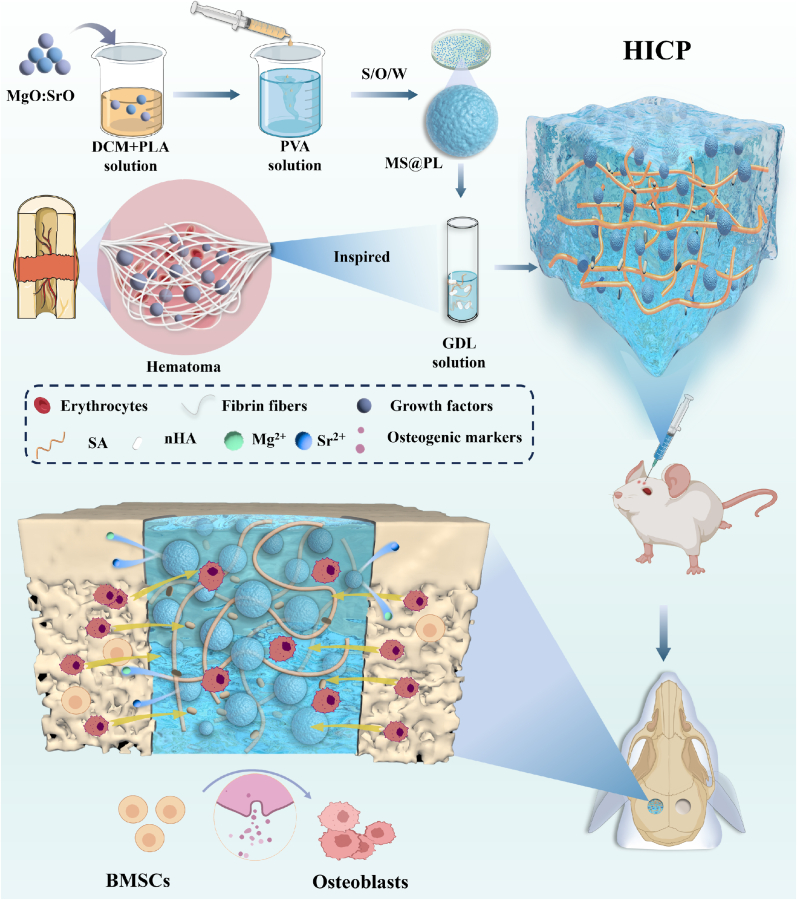
Schematic illustration of preparation of the hematoma-inspired injectable composite hydrogel (HICP) to promote osteogenic differentiation of BMSCs and facilitate the repair of critical-size cranial defects in rats.

Hematoma that contains accumulated blood and coagulation components plays the essential role in the initial stage of the physiological bone healing process [23,24]. It not only releases various growth factors for cell recruitment, migration, and differentiation to promote bone healing, but also serves as a temporary scaffold to support bone healing [25]. Herein, we engineered a hematoma-inspired injectable composite hydrogel (HICP) to exert sustained osteogenic effects for treating critical-sized bone defects (Fig. 1). Osteogenic polylactide microspheres simultaneously embedded with magnesium oxide (MgO) and strontium oxide (SrO) were first achieved and exhibited the rapid release of Sr^2+^ and a gradual release of Mg^2+^ to maintain the long-term high-level concentration of bioactive ions. Subsequently, encapsulation of osteogenic microspheres in the alginate hydrogel was implemented through crosslinking by slowly released Ca^2+^ from insoluble nano-hydroxyapatite (nHA). The resultant HICP exhibited favorable injectability, high mechanical strength and good biocompatibility. *In vitro* experiment validated that HICP promoted proliferation, migration and osteoblastic differentiation of BMSCs as well as calcium deposit. As demonstrated by an *in vivo* rat calvarial defect model, the application of HICP displayed exceptional ability in facilitating new bone regeneration within the defect area. The current work opens a valuable avenue to develop advanced osteogenic hydrogels for critical-sized bone defect regeneration.

## 2 Materials and methods

### 2.1 Materials

Polylactide (PLA, LX175, *M*_w_ = 1.88 × 10^5^ g/mol) was provided by Purac-Biochem B.V., Netherlands. MgO and SrO were obtained from Macklin, China. Polyvinyl alcohol (PVA, 1788, *M*_w_ = 4 × 10^4^ g/mol) and dichloromethane (DCM) were supplied by Chengdu Kelong Chemical Reagent Factory, China. D-Glucono-δ-lactone (GDL) and sodium alginate (SA) were purchased from Shanghai Macklin Biochemical Co., Ltd (China). Nano-hydroxyapatite (nHA, rod-shaped purity >99 %) was supplied from Suzhou Beike Nano Materials Co., Ltd (China).

### 2.2 Preparation of the composite hydrogel

The osteogenic microspheres encapsulated with MgO and SrO were first fabricated by a modified solid-in-oil-in-water (S/O/W) emulsion method. PLA granules were dissolved in DCM to obtain a PLA/DCM solution with the concentration of 5 % w/v as the oil phase. MgO and SrO in a weight ratio of 1:1 were homogenously dispersed in the above oil phase. And their additive amount was 20 wt%. The suspension was subsequently dropped into the PVA aqueous solution (0.8 % w/v) and stirred for 8 h at 35 °C to evaporate DCM. The osteogenic microspheres (MS@PL) were finally obtained by collecting *via* Buchner funnel, washed with deionized water and dried in a vacuum oven. Unless otherwise stated, the total additive amount of MgO and SrO in the microspheres was 20 wt%. For comparison, other microspheres were prepared and named as PL (no loading), Mg@PL (loaded with MgO), and Sr@PL (loaded with SrO). 0.2 g of MS@PL and 0.6 wt% of nHA were uniformly dispersed in a 2 % w/v SA solution (20 mL), followed by adding 5 g of GDL. The composite hydrogel embedded osteogenic microspheres were spontaneously formed and termed as HICP. Composite hydrogels containing 0 and 0.3 wt% hydroxyapatite were synthesized and designated as 0HICP and 0.3HICP, respectively. Additionally, a composite hydrogel with 0.6 wt% hydroxyapatite in the absence of MS@PL was labeled as 0.6SA.

### 2.3 Characterizations

The surface morphology of osteogenic microspheres was observed by a field-emission scanning electron microscope (FE-SEM, Nova NanoSEM450, FEI, USA). The chemical composition of microspheres was examined by an energy-dispersive spectroscopy (EDS, X-Max Extreme detector, OXFORD, UK) and X-ray photoelectron spectroscopy (XPS, AXIS Supra, Kratos, UK). X-ray diffraction (XRD, Ultima IV, Rigaku, Japan) was performed to identify MgO and SrO in the microspheres. The thermogravimetric analysis (TGA, TG 209 F1 Libra, NETZSCH, Germany) was conducted to measure the additive amounts of metal oxides at a heat rate of 10 °C/min. The compressive strength of the composite hydrogel (diameter = 15 mm) was tested using a universal testing machine (model 5967, Instron, USA) at a rate of 1 mm/min. Cyclic compression tests of the hydrogel were conducted at compressive strains of 20 % and 30 % with 15 cycles for each strain level. The storage modulus and loss modulus of the hydrogel were measured using a rotational rheometer (model MCR302, Anton-Paar, Austria). Dynamic oscillatory tests were conducted with a fixed strain of 1 % and angular frequency sweeps from 0.01 to 100 rad/s.

### 2.4 In vitro release of MgO and SrO

The release of Mg^2+^ and Sr^2+^ from osteogenic microspheres (30 mg) was executed in phosphate-buffered saline (PBS) solution (10 mL) at 37 °C for four weeks. The suspension was filtered and collected at predetermined intervals, followed by the addition of hydrochloric acid (2 mL) to dissolve MgO and SrO. Inductively coupled plasma optical emission spectrometry (ICP-OES, Agilent 5100 SVDV, USA) was performed to determine the concentrations of Mg^2+^ and Sr^2+^. Subsequently, fresh PBS solution (10 mL) was utilized to resuspend microspheres for the next measurement. The entire release of Mg^2+^ and Sr^2+^ in the osteogenic microspheres (30 mg) was set as the reference line. Every sample was assessed for three times at least.

### 2.5 In vitro biomineralization evaluation

The osteogenic microspheres were immersed in the two-fold simulated body fluid (2SBF) as the mineralization solution, prepared by dissolving multiple inorganic salts (NaCl, NaHCO_3_, Na_2_SO_4_, KCl, K_2_HPO_4_, MgCl_2_, and CaCl_2_) and buffered to the appropriate pH of 7.4 using Tris-HCl. After incubation for 7 days, the microspheres were collected and gently rinsed with PBS. SEM and EDS mapping were employed to observe the morphology and elemental distribution of the mineralized microspheres.

### 2.6 Biocompatibility

BMSCs were selected as test cells and plated at 1 × 10^5^ cells per well. The composite hydrogels sterilized by UV irradiation were co-cultured with BMSCs in the *α*-MEM medium for 1, 3, and 7 days. The Cell Counting Kit-8 (CCK-8) assay was applied to each well, and the absorbance at 450 nm was measured by a microplate reader (Infinite M200 Pro, Tecan, Switzerland). Live-dead cell staining was conducted using an inverted fluorescence microscope (Ti-2u, Nikon, Japan) to evaluate the cell viability.

### 2.7 Immunohistochemical staining

BMSCs were seeded into 24-well plates with 4 × 10^4^ per well, incubated with the composite hydrogel for 7 days and then fixed with 4 % paraformaldehyde for 20 min. After removing the fixation solution, 0.1 % Triton X-100 was added followed by washing with PBS solution. The primary and secondary antibodies targeting RUNX-2 and Ki-67 were sequentially added for incubation. The DAPI solution was used to stain the cell nuclei. The staining patterns for RUNX-2 and Ki-67 were visualized and imaged using a confocal microscope (LSM700, Zeiss, Germany). On day 14 of BMSCs co-culture with the composite hydrogel, mineralized nodules were identified. BMSCs were incubated at room temperature for 15 min with a 2 % Alizarin Red S (ARS) solution. The cells were then washed with distilled water, and mineralized nodules were observed by a standard optical microscope.

### 2.8 In vitro osteogenic differentiation

After day 7 of BMSC culture, cells were lysed to extract the proteins that were placed on SDS-PAGE and transferred to a polyvinylidene fluoride membrane. The membrane was immersed in blocking solution overnight at 4 °C. Subsequently, the membrane was incubated with primary antibodies against RUNX2, OCN, Wnt3 and β-catenin protein. Then, horseradish peroxidase-conjugated secondary antibody was added and allowed to react at 37 °C for 1 h after washing with TBST buffer, and color development was performed through chemiluminescence.

### 2.9 In vivo bone regeneration evaluation

#### 2.9.1 In vivo critical-size cranial defect model

The *in vivo* experiments received ethical approval from the Experimental Animal Ethics Committee of Guangzhou Medical University (Ethics approval number: S2022-108). Sixteen male SD rats were obtained from Guangzhou Ruige Biotechnology Co., Ltd. (License No.: SCXK2021-0059) and randomly divided into four groups (*n* = 4): the alginate hydrogel embedded PLA microspheres (PS), the alginate hydrogel loaded with MgO/PLA microspheres (MPS), the alginate hydrogel embedded SrO/PLA microspheres (SPS), and hematoma-inspired injectable composite hydrogels (HICP) groups. After anesthesia induction via intraperitoneal injection of 10 % chloral hydrate, the skull was exposed by cutting the skin and periosteum layer by layer, and a cylindrical defect with a diameter of 5 mm was drilled bilaterally along the sagittal suture using a hollow drill bit. The composite hydrogels were injected into the defects followed by wound suturing.

#### 2.9.2 Ultrasound examination of cranial defect and histology

Following a 4-week implantation period, the rats (n = 4) were euthanized humanely under anesthesia. The cranial tissues were then collected, and cranial injuries along the coronal plane were evaluated using an ultrasonic device (Navi, Wisonic, China) in the bone tissue detection mode.

Skull tissues were decalcified in ethylenediaminetetraacetic acid solution (EDTA) for 4 weeks. The samples were embedded in paraffin and sectioned to a thickness of 5 μm at the center of the cranial bone. Hematoxylin and eosin (H&E) and Masson's trichrome staining were utilized to evaluate bone regeneration and collagen deposition. Immunohistochemical staining for ALP, Tubulin, and GSK-3 was performed to assess the osteogenic potential of the composite hydrogel. Their relative expression was calculated by the ImageJ software.

#### 2.9.3 Western blot analysis

The protein was extracted from the cranial bone tissue by cell lysis buffer and separated by SDS-PAGE, followed by transfer to a polyvinylidene fluoride film. The membrane was immersed in blocking solution overnight at 4 °C. The membrane was then washed and incubated with primary antibodies: ALPP, Ang-1, OCN, OPN, OSX, and RUNX2 at 4 °C for 12 h. Subsequently, the membranes were then washed three times with TBST buffer and incubated with a horseradish peroxidase-conjugated secondary antibody for 1 h at 37 °C. Protein expression levels were quantified via chemiluminescent detection. Band intensities (gray values) of target proteins were measured using ImageJ software and normalized to GAPDH as an internal control.

### 2.10 Statistical analysis

All data were expressed as the mean ± standard deviation. Statistical comparisons among multiple groups were performed by using one-way or two-way ANOVA. Values were considered statistically significant at *p <* 0.05.

## 3 Results and discussion

### 3.1 Preparation and characterization of osteogenic microspheres

**Fig. 2:**
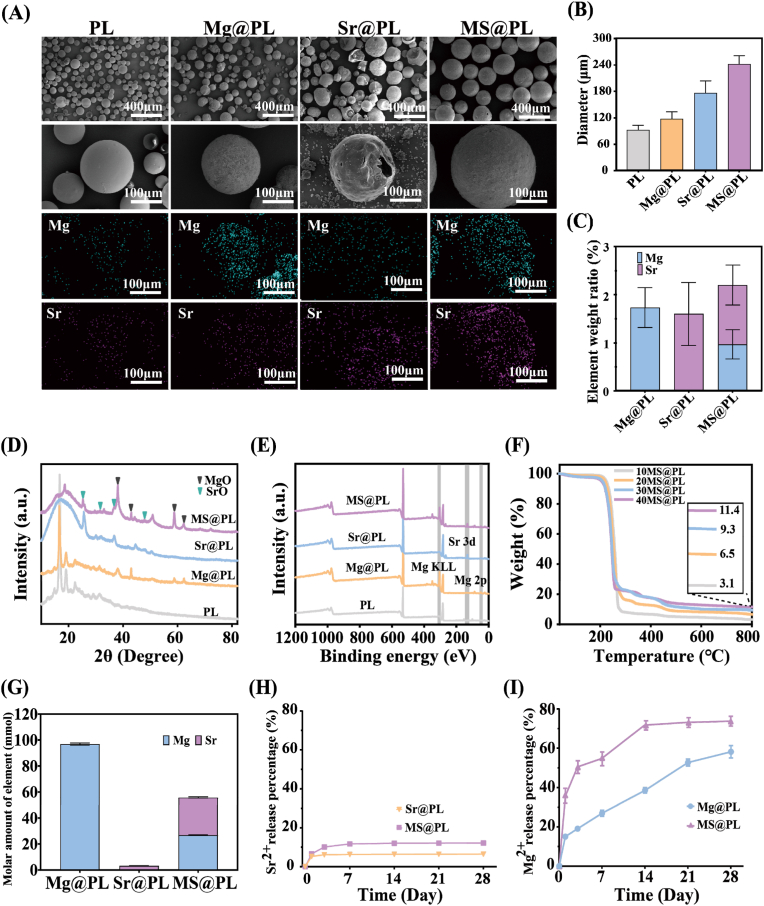
Morphology, composition and ion release of osteogenic microspheres. (A) SEM images and EDS mapping of microspheres. (B) Average diameter, (C) Element content, (D) XRD curves and (E) XPS spectra of microspheres. (F) TGA curves of MS@PL with different contents. (G) Mg^2+^ and Sr^2+^ contents of Mg@PL, Sr@PL and MS@PL as measured by ICP-OES. The cumulative release percentages of (H) Sr^2+^ and (I) Mg^2+^ from osteogenic microspheres over time in PBS solution at 37 °C.

The osteogenic microspheres embedded metallic oxides were fabricated by an S/O/W emulsion-solvent evaporation method. Microstructure observation reveals that the average size of polyhedral MgO and spindly SrO is 195.5 and 627.6 nm (Fig. S1). Osteogenic microspheres present relatively rough surfaces after incorporating metallic oxides, compared with the smooth surface of pure PL (Fig. 2A). The metallic oxides are dispersed in the oil phase containing the dissolved PLA chains and form stable emulsion droplets in the outer aqueous phase. During the evaporation of the organic solvent, the mixture consisting of metallic oxides and PLA chains shrinks and solidifies into osteogenic microspheres. The average diameter of PL is 92 μm, and a significant increase of the mean diameter is perceived for osteogenic microspheres with metallic oxides. Especially, MS@PL exhibits good sphericity with an average diameter of 242 μm (Fig. 2B and S2). EDS mapping images manifest that Mg and Sr elements are newly detected in Mg@PL and Sr@PL respectively, implying the encapsulation of MgO and SrO. The surface of MS@PL displays the uniform distribution of Mg and Sr elements, indicative of the homogenous dispersion of MgO and SrO. The total contents of Mg and Sr elements in MS@PL are comparable to that in Mg@PL and Sr@PL respectively (Fig. 2C).

Fig. 2D shows that additional characteristic peaks of MgO (38.1°, 42.9°, 58.8° and 62.3°) and SrO (25.3°, 31.6°, 36.8° and 47.8°) are perceptible in the Mg@PL and Sr@PL respectively, compared to PL. The XRD profile of MS@PL presents both the diffraction peaks of MgO and SrO, suggesting the successful incorporation of hybrid metallic oxides. Further evidence was accessed by XPS spectra. For the XPS spectrum of MS@PL, the peaks at 48.1 and 304.1 eV are assigned to Mg 2p and Mg KLL, and the peaks at 131.3 and 132.8 eV are related to Sr 3d (Fig. 2E). TGA analysis shows that the residual mass of 40MS@PL rises to 11.4 wt% from 3.1 wt% for 10MS@PL. The additive amounts of metallic oxides in osteogenic microspheres can be readily regulated by controlling the original feedings (Fig. 2F). The practical loading of MgO and SrO in osteogenic microspheres is further measured by analyzing the release concentration of Mg^2+^ and Sr^2+^ (Fig. 2G). The molar ratio of Mg and Sr in MS@PL maintains nearly the same. And MS@PL exhibits the higher Sr content compared to Sr@PL, ascribed to the leakage of nanoscale SrO caused by the surface breakage and even indentation of Sr@PL.

The release of bioactive ions plays a crucial role in exerting the biological activity of osteogenic microspheres. Fig. 2H and I present that continuous release of metallic ions is detected for all osteogenic microspheres loading with metallic oxides within 28 days, after an incipient burst release on day 1. The cumulative release percentage of Sr^2+^ of MS@PL rapidly reaches up to 10.08 % during 3 days and increases to 12.14 % on day 28. By contrast, the release of Mg^2+^ increases slowly in MS@PL, supplementing the Sr^2+^ release and offering sustained biological benefits during the entire repair period (Fig. S3). SEM observation after full release of metal ions exhibits MS@PL displays a certain amount of cavities after completely releasing metal ions (Fig. S4). Noteworthily, the rapid release of Sr^2+^ and slow release of Mg^2+^ forms the complementary release of bioactive metallic ions to keep the appropriate concentration of therapeutic metal ions and provide a favorable osteogenic microenvironment. Furthermore, it circumvents the potential risks associated with excessive use of single metal ion [26,27].

### 3.2 In vitro biomineralization of osteogenic microspheres

**Fig. 3:**
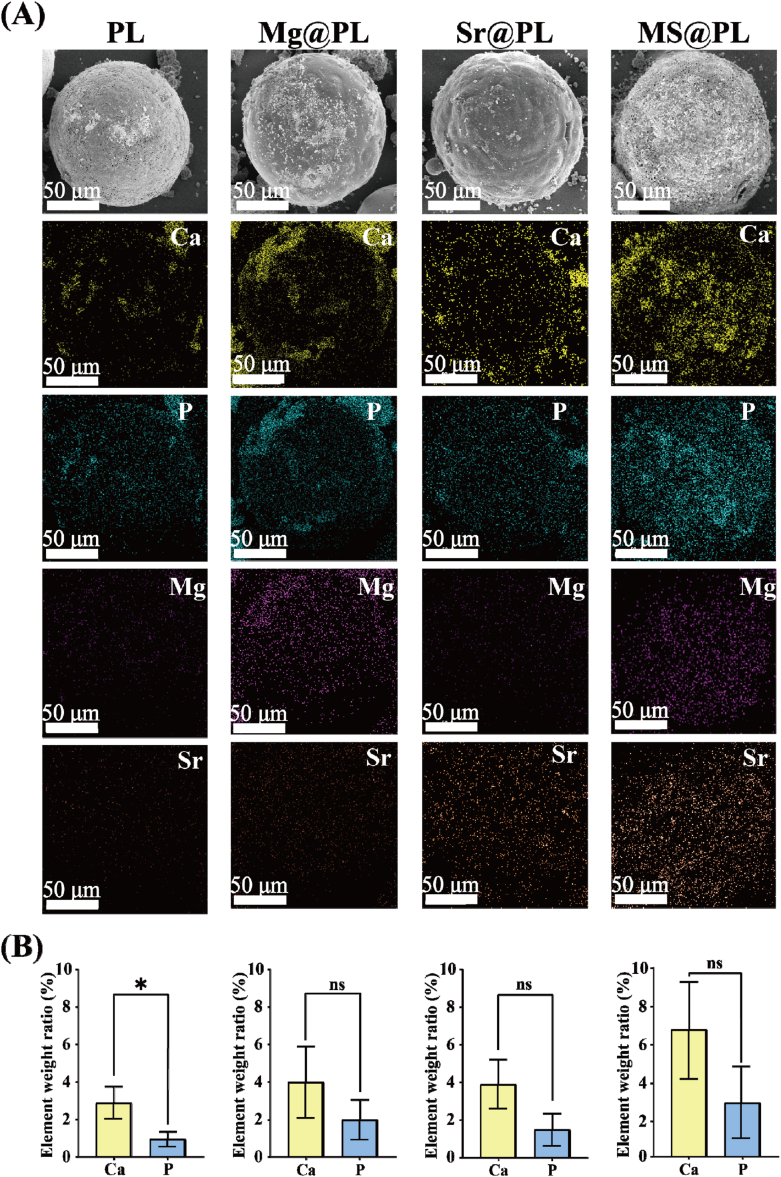
*In vitro* mineralization of osteogenic microspheres and biocompatibility of the composite hydrogel. (A) SEM images and EDS mapping of mineralized microspheres. (B) The element weight ratio of the Ca and P of mineralized sediments for osteogenic microspheres.

Mineralization constitutes a crucial stage in bone formation and serves as a key element in facilitating bone repair and regeneration [28,29]. The mineralization process involves the localized accumulation of inorganic components (Ca^2+^, PO_4_^3−^) as bioactive ions for osteogenic differentiation [30]. Meanwhile, mineralization facilitates the formation of mineralized collagen fibrils to guide the orchestrated growth of osteoblasts and provide structural reinforcement for bone formation [31,32]. The osteogenic microspheres are dipped in 2SBF for 7 days to evaluate their *in vitro* biomineralization property, closely related to the potential for facilitating bone regeneration. Only bits of sediment appear on the PL surface, while significantly increasing sediments are observed on the surface of all osteogenic microspheres embedded with metallic oxides. The uniform and abundant calcium (Ca) and phosphorus (P) elements confirm the formation of Ca and P compounds on surfaces of osteogenic microspheres (Fig. 3A). The ratio of Ca and P of mineralized sediments for osteogenic microspheres is approximated to that of HA, maximizing the simulation of the chemical composition of natural bone to promote new bone formation (Table S1). Quantitative results show that MS@PL displays the highest element content of Ca (6.69 %) and P (2.85 %), 131 % and 197 % higher than those of PL (Fig. 3B). It reveals that MS@PL possesses the strongest ability to induce biomineralization benefiting from the synergistic osteogenesis of Mg^2+^ and Sr^2+^. The rapid release of Sr^2+^ and the gradual release of Mg^2+^ guarantee the consistent high-concentration bioactive ions to effectively promote biomineralization [33].

### 3.3 Injectability, swelling capacity, mechanical property and biocompatibility of composite hydrogels

**Fig. 4:**
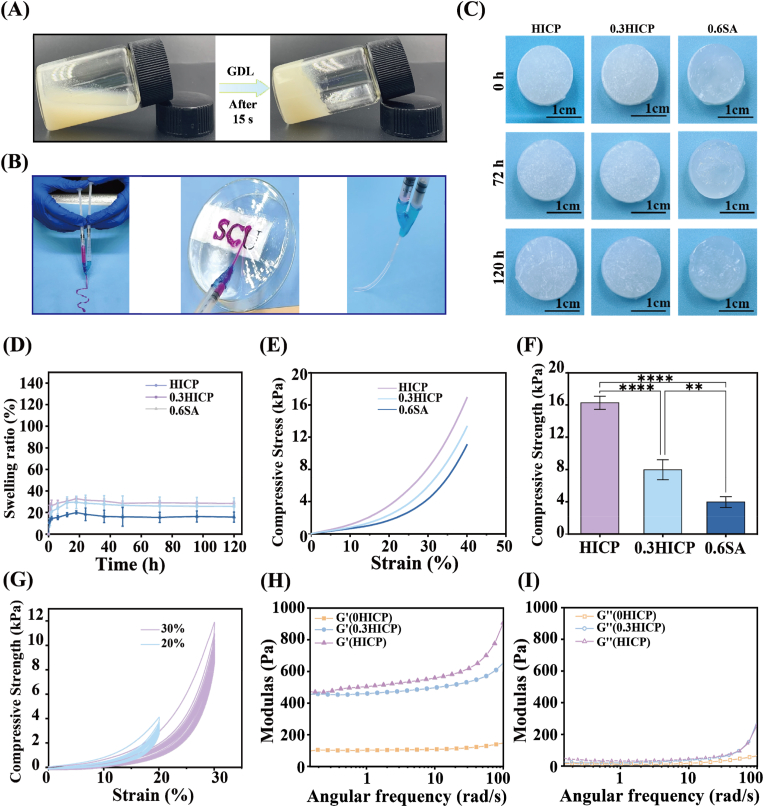
Injectability, swelling capacity and mechanical property of composite hydrogels. Photographs of (A) the sol-gel transition and (B) injectability from a syringe of the composite hydrogel. (C) Optical images of the swelling composite hydrogels and (D) the relevant swelling ratio as a function of time. (E) The compressive stress-strain curves and (F) the compression strength of the hydrogels. (G) The cyclic compressive curves of HICP at different strains. (H) Frequency sweep of *G*′ and *G*″ of HICP with different nHA contents.

The injectability and *in situ* molding of hydrogels are urgently required for administration in a minimally invasive approach and for defect conformation of irregular bone defects. Alginate hydrogels are generally crosslinked by divalent metal ions (Ca^2+^, Zn^2+^, Sr^2+^) at the rapid gelation rate, severely compromising the injectability of hydrogels [34]. In order to overcome the above predicament, we propose that the slow release of Ca^2+^ from insoluble nHA is achieved by the hydrolysis of GDL to crosslink SA and complete the sol-gel transition process. The composite alginate hydrogel embedded osteogenic microspheres is gelled within 15 s (Fig. 4A). The prolonged gelation time enables the hydrogel to be extruded by the home-made dual-component syringe and molded into the solid structure of the “SCU” word (Fig. 4B). Therefore, the composite hydrogels are able to be feasibly injected and adapt to bone defects with complex shapes and are not readily lost from bone defects after injection. Osteogenic microspheres exhibit the uniform distribution in the alginate hydrogels to provide the structural basis for guaranteeing the homogeneous and continuous release of the bioactive Mg^2+^ and Sr^2+^ in the 3D space to promote new bone formation (Fig. S5).

The swelling capacity of hydrogels is relatively important for the application in bone tissue engineering [35]. As shown in Fig. 4C–D, there is no apparent variation of geometric parameters for all composite hydrogels after being immersed in PBS for 120 h. The swelling ratio of hydrogels rapidly rises at the first 18 h, followed by a gradual increase towards the swelling equilibrium. The introduction of MS@PL into HICP leads to a higher swelling ratio (29.0 %) than that of 0.6SA (15.6 %). The augmented swelling behavior reveals the potential of HICP in bone defect repair, especially in osteoporotic bones, where it can efficiently fill the intricate geometric bone defects by absorbing bodily fluids. The enhancement of the nHA content from 0.3 to 0.6 wt% has an ignorable influence on the swelling ratio of HICP. The 0HICP without hydroxyapatite present the poor structural integrity and rapidly disintegrated after immersing in the PBS solution for 1 h. It is noted that the swelling ratio of the composite hydrogels is relatively low (<35 %), avoiding the potential damage for new bone tissues caused by the excessive volume of hydrogels.

Robust mechanical properties are of the essence for bone repair materials to sustain physiological stresses while retaining the intact structure. The compressive stress-strain profiles show that the incorporation of osteogenic microspheres ameliorates compression strengths of HICP compared to 0.6SA. The enhancement of the nHA content is beneficial to improve the mechanical strength of HICP. The compression strength of HICP is up to 17.15 kPa, 86.2 % and 30.1 % higher than that of 0.3HICP and 0.6SA (Fig. 4E–F). The addition of osteogenic microspheres fills part of the pore structure of the hydrogel network and facilitates the stress transfer. The cyclic compressive tests confirm the favorable fatigue stability of HICP supported by the almost overlapping compression curves (Fig. 4G). The dynamic rheological behavior of HICP was measured to evaluate its mechanical performance and viscoelasticity. Fig. 4H–I shows that the storage modulus (*G*′) consistently exceeds the loss modulus (*G*″) of HICP with different nHA contents, indicating the elastic behavior and stable network structure of HICP. The increase of the nHA content improves the *G*′ and *G*″of HICP, ascribed to the more perfect hydrogel network.

**Fig. 5:**
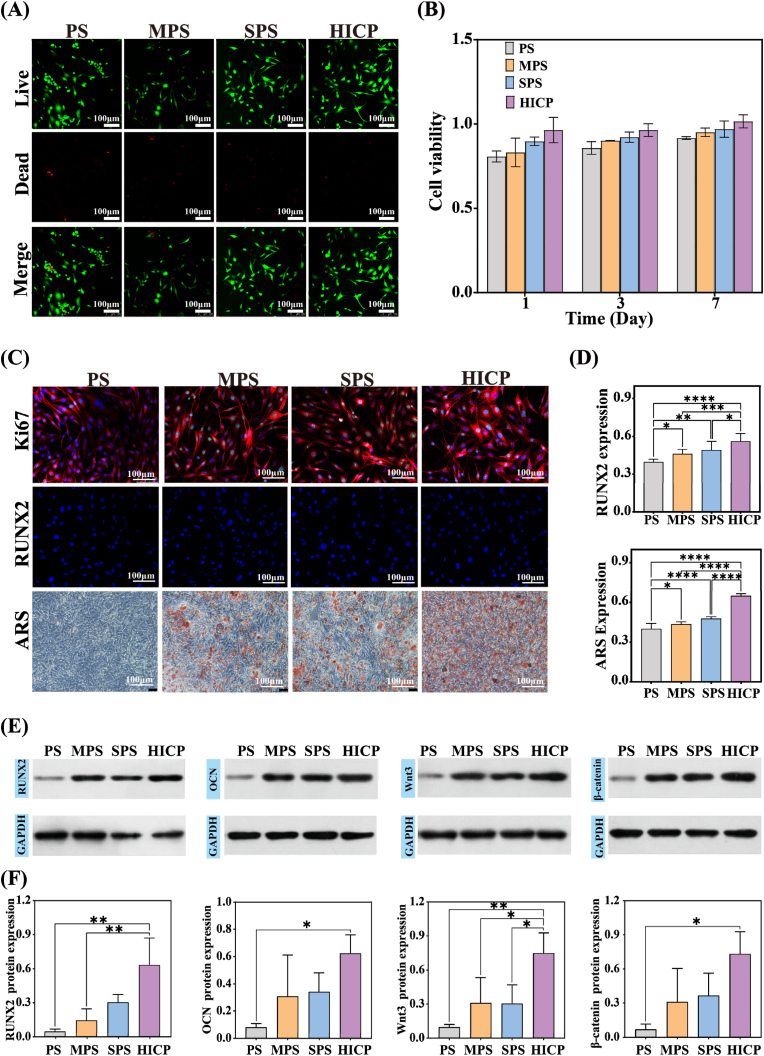
*In vitro* osteogenic differentiation of BMSCs induced by the composite hydrogels. (A) Live/dead staining of BMSCs on day 3. (B) The BMSC viability as a function of incubation time. (C) The staining of Ki67, RUNX2 and ARS. The expression intensity of (D) RUNX2 and ARS measured by the staining images. (E) Protein bands assessed by western blotting analysis and (F) quantitative protein expression levels for RUNX2, OCN, Wnt3 and β-catenin.

Biocompatibility is a crucial precondition for composite hydrogels in osteogenic biomedical applications [36,37]. Live/dead staining images exhibit the majority of the BMSCs are stained in green (live cells) and almost no visible cells are stained in red (dead cells), after cocultured with composite hydrogels for 3 days. Quantitative CCK-8 results validate that ongoing cell growth is perceived with the extension of incubation time (Fig. 5A–B and S6). The synchronous introduction of MgO and SrO prominently promotes the proliferation of BMSCs after co-culturing with HICP. Unluckily, there is no obvious positive effect on facilitating cell growth after the incorporation of single metal oxide in MPS and SPS. *In vitro* hemolysis test reveals that the hemolysis ratios of all composite hydrogels range from 1.3 to 2.7 %, obviously lower than the safety threshold of 5 % according to the ISO 10993-4 guideline (Fig. S7). These findings confirm the good biocompatibility of the composite hydrogels. The cell scratch assay indicates that HICP markedly promotes the cell migration of BMSCs and the scratch closure. The quantitative cell migration rate of the HICP group is up to 83.9 %, significantly superior to the PS (18.2 %), MPS (38.6 %) and SPS (64.7 %) groups (Fig. S8). The accelerated migration of BMSCs actuated by HICP is expected to promote new bone formation.

### 3.4 In vitro osteogenic differentiation of composite hydrogels

The osteogenic induction capacity of the composite hydrogels determines the repair process of bone defects [38]. We first investigated the influence of hydrogels on the proliferation of BMSCs. Ki67 is a nuclear antigen associated with cell proliferation that specially targets proliferating nuclei and is utilized as a valuable biomarker for assessing the proliferative activity of BMSCs. The deeper Ki67 staining and larger stained area for MPS, SPS and HICP confirm that the incorporation of metallic oxides is conducive to the proliferation of BMSCs compared to PS (Fig. 5C). RUNX2 is a crucial transcription factor that initiates and regulates osteoblastic differentiation. After 24 h of osteogenesis induction, HICP exhibits the most extensive and intense RUNX2 staining than the other three composite hydrogels, indicating the strong capacity of promoting osteoblastic differentiation. Calcium nodule formation is regarded as a late-stage hallmark for osteogenesis. Alizarin Red S (ARS) staining was conducted to detect the mineralization potency of the composite hydrogels. Almost no visible calcium modules were found for PS, while the deposition area of calcium nodules augments for composite hydrogels with metallic oxides. Especially, the distribution intensity of calcium nodules for HICP is 0.650, much higher than that of PS, MPS and SPS (Fig. 5C–D).

To further elucidate the potential of composite hydrogels to facilitate osteogenic differentiation of BMSCs, western blot analysis was carried out to measure the expression level of OCN and RUNX2 as key osteogenic markers in BMSCs. As presented in Fig. 5E and F, cells treated with HICP have the highest expression of the above osteogenic-related proteins compared to PS, MPS and SPS. Specifically, the OCN protein expression level for HICP was upregulated by 690 %, 102 % and 83 % compared to PS, MPS and SPS respectively. The similar trend is observed in the expression of RUNX2 for HICP. These results imply that HICP possesses a more pronounced promotion of osteogenic differentiation in BMSCs when synchronously incorporating Mg and Sr components. The underlying mechanism of promoting osteogenesis for HICP was further investigated and revealed. The Wnt/β-catenin pathway is a key signaling pathway about the differentiation of BMSCs into osteoblasts. The expression of Wnt3 and β-catenin apparently increases for HICP compared with PS, MPS and SPS. For instance, the expression of the Wnt3 protein is increased by 675 %, 143 %, and 147 % compared with PS, MPS and SPS. It confirms that the addition of MgO and SrO generates the synergistic effect on activating the Wnt/β-catenin signaling pathway and facilitate osteogenic differentiation.

### 3.5 In vivo osteogenic bone regeneration evaluations

**Fig. 6:**
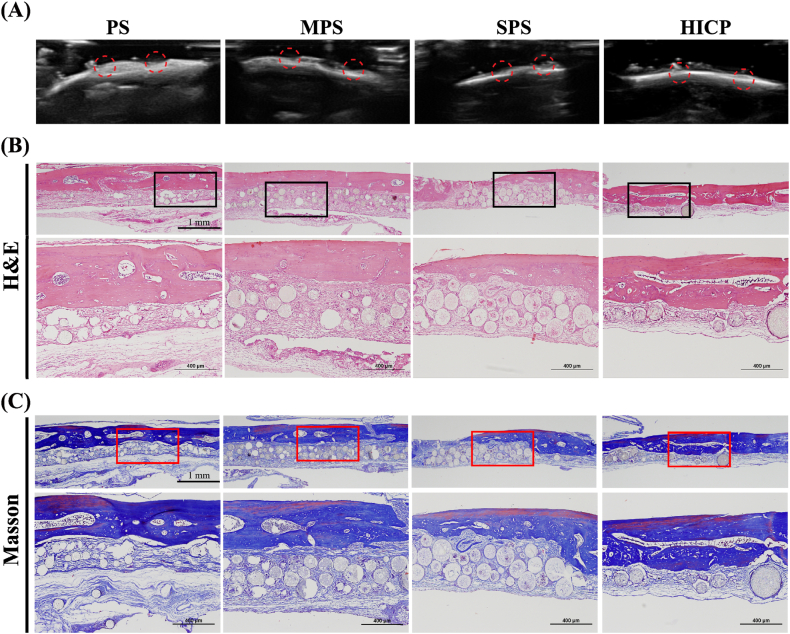
*In vivo* bone regeneration in a rat cranial defect model. (A) Ultrasound images of the skull defect at 4 weeks. (C) H&E staining and (D) Masson's trichrome staining of the cranial defects at 4 weeks.

To investigate the therapeutic efficacy of composite hydrogels for bone defect repair *in vivo*, we created critical-sized cranial defects (diameter = 5 mm) in SD rats. The hydrogels were directly and easily injected into the defect site for 4 weeks. Ultrasound analysis offers the information about the bone structure, quality and density to assess the bone repair status and identify conditions about the bone defects or non-union. The bone repair effect of composite hydrogels with metallic oxide groups is superior to that of the PS group (Fig. 6A). A depressed cranial bone injury and interruptions at the depressed area in the high-density cortical bone are observed in the PS group. Furthermore, the concave area is filled with low-density microspheres but no signs of bone healing are detected. In contrast, low-density shadows inside the injury and the presence of cortical bone lines above the depression are perceived for the MPS and SPS groups, suggesting the certain healing of cranial bone injuries and manifesting the positive osteogenic effect of introducing MgO or SrO. The healing of concave cranial bone injuries is obviously observed in the HICP group, and complete and continuous cortical bone lines as well as no signs of protrusion or sinking are visible. This finding reveals that HICP exhibits the exceptional osteogenic effect compared with other composite hydrogels, owing to the synergistic release of Mg^2+^ and Sr^2+^ that stimulates the differentiation of BMSCs and suppressing the osteoclastic activity.

H&E and Masson staining further confirm the formation of new bone and the maturation of new bone tissue histologically (Fig. 6B and C). Stained sections show no significant inflammatory response in any groups. The PS group presents that a thin layer of the fibrous tissue mainly bridges the bone defect area and no apparent signs of new bone regeneration appear. For the MPS and SPS groups, the infiltration and growth of fibrous tissues are decreased, and abundant collagen-rich extracellular matrix fills the gaps between composite hydrogels. Notably, the richest collagen content and bone marrow-like structure are found in the HICP group. After implanting into the bone defect for 4 weeks, all composite hydrogels are not completely degraded. The slow degradation of our hydrogels guarantees the long-term release of bioactive metallic ions to promote the bone defect repair and avoid the compromise of osteogenic effects caused by the premature degradation of hydrogels. Different from PS shown in the staining image, all the MPS, SPS and HICP themselves are visually stained by H&E and Masson reagents. This finding indicates that the degradation of the composite hydrogels with metallic oxides promotes the infiltration of cells and osteanagenesis.

Encapsulating functional nanoparticles into hydrogels to promote bone defect repair is an attractive strategy for preparing osteogenic materials. Jiang et al. directly introduced graphene oxide sheets in alginate/sericin hydrogels to enhance the spreading, osteogenic differentiation, and mineralization of BMSCs for the bone generation [38]. Zhou et al. prepared asymmetric Mg@polyethylene glycol microspheres embedded in poly (lactic-co-glycolic acid) hydrogels to exert immunomodulatory effects such as reducing intracellular reactive oxygen species, and guiding macrophage polarization toward the M2 phenotype to promote osteoporotic bone repair [39]. Xiao et al. introduced exfoliated Ti_3_C_2_T_x_ and hydrothermal carbon dots in the gelatin hydrogel to kill bacteria and enhance the osteogenic activity respectively for facilitating infected bone defect repair [40]. In contrast, we prepared PLA osteogenic microspheres that simultaneously embedded MgO and SrO by a simple and scalable solid-in-oil-in-water emulsion method. The alginate hydrogel was further employed to encapsulate PLA osteogenic microspheres and achieve injectability by slow release of Ca^2+^. The sequential yet complementary release of rapidly released Sr^2+^ and gradually released Mg^2+^ in our composite hydrogels contributes to maintain long-term high-level concentrations of bioactive ions to effectively promote critical-sized bone defect repair.

**Fig. 7:**
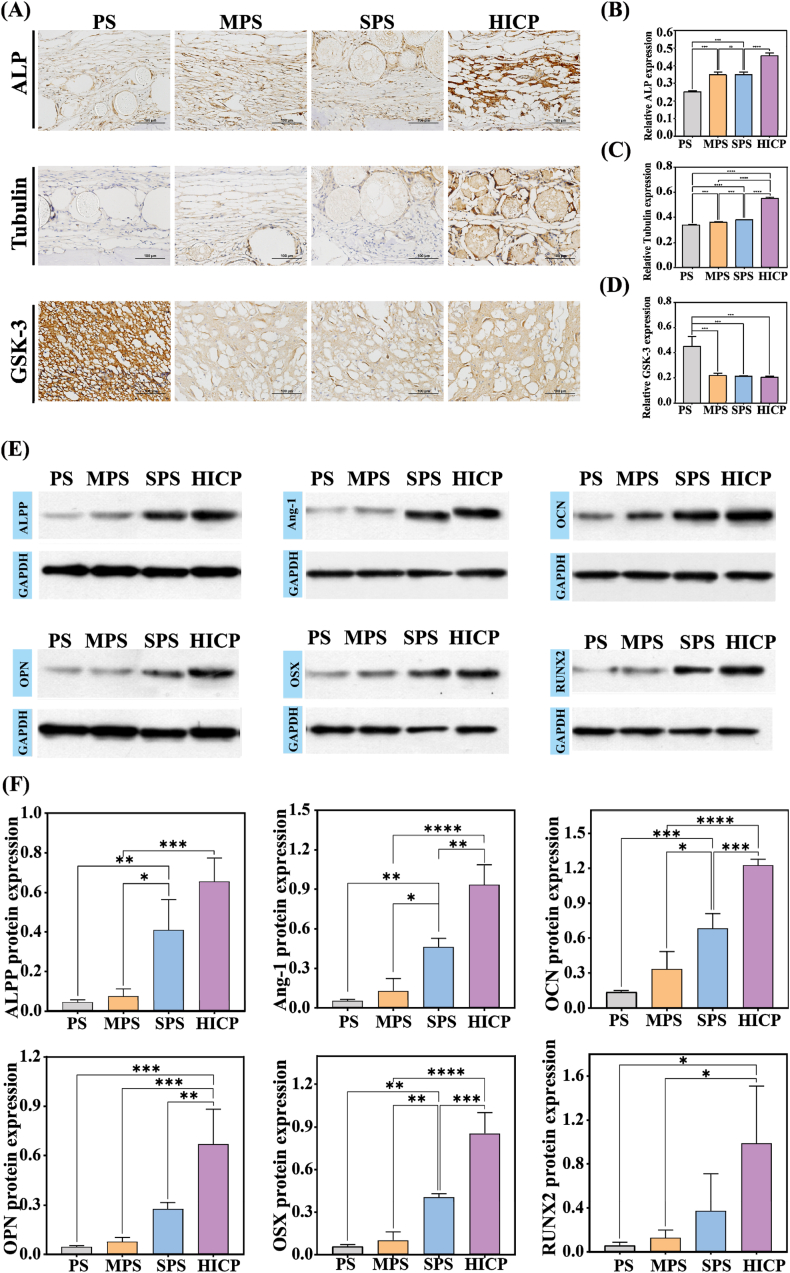
Immunohistochemical staining and western blot analysis of regenerated bone tissues. (A) Immunohistochemical staining of ALP, Tubulin and GSK-3 at 4 weeks. The quantitative relative expression of (B) ALP, (C) Tubulin and (D) GSK-3. (E) Protein bands assessed by western blotting analysis and (F–K) quantitative protein expression levels for ALPP, OCN, OSX, Ang-1, OPN and RUNX2.

Given the importance of osteogenic and remodeling markers on the repair process of bone defects, immunohistochemical staining was performed to analyze the expression of key proteins. By 4 weeks, the composite hydrogel with metallic oxide groups exhibit larger staining areas of ALP and Tubulin compared with the PS group (Fig. 7A–C). Notably, the HICP group displays the largest staining area. For instance, the relative expression of ALP is 0.457 in the HICP group, 81.3 %, 31.0 % and 28.8 % higher than that of the PS, MPS and SPS groups respectively. Moreover, the HICP group shows the smallest staining area of GSK-3 with a relative expression of 0.206, 54.4 % lower than that of PS (Fig. 7D). Western blot analysis was further conducted to determine the protein expression of osteogenic factors (ALPP, OCN, OSX, Ang-1, OPN, and RUNX2) in the regeneration tissues (Fig. 7E). The osteogenic factor proteins in postoperative tissues treated with composite hydrogels with metallic oxides exhibit observably higher expression compared to the PS group. In particular, the HICP group displays the highest expression level (Fig. 7F). These results collectively underscore the high effectiveness of HICP in accelerating *in situ* bone regeneration, positioning it as a promising candidate for therapeutic application in the bone defect repair.

## 4 Conclusion

In summary, we engineered a hematoma-inspired injectable composite hydrogel with hybrid metal ion microspheres to accelerate bone regeneration in critical-sized defects. Osteogenic microspheres incorporating MgO and SrO were first fabricated and exhibited the complementary release of Mg^2+^ and Sr^2+^ to maintain suitably high concentration of bioactive ions over the bone repair time. The alginate hydrogel embedded osteogenic microspheres and crosslinked by the slow release of Ca^2+^ from insoluble nHA showed the exceptional injectability, high mechanical strength and good biocompatibility. *In vitro* experiments confirmed that the resultant composite hydrogel promoted the proliferation and osteogenic differentiation of BMSCs as well as the upregulation of osteogenesis-related proteins. After implantation of the hematoma-inspired injectable composite hydrogel, the process of bone regeneration was prominently expedited within the critical-sized bone defects owing to the synergistic release of bioactive metallic ions. This work provides a promising alternative material for delivering therapeutic ions to enhance bone regeneration.

## CRediT authorship contribution statement

**Fan Cao:** Writing – original draft, Methodology, Investigation. **Jin-Yong Wu:** Writing – original draft, Validation, Methodology. **Qing-Ning Wang:** Visualization, Methodology. **Jun-Jie Xiao:** Visualization, Validation. **Zhu Chen:** Visualization. **Ya-Wen Wang:** Validation, Methodology. **Zhi-Guo Wang:** Writing – review & editing, Visualization, Methodology, Conceptualization. **Jia-Zhuang Xu:** Supervision, Funding acquisition. **Zhong-Ming Li:** Supervision, Project administration. **Bai-Song Zhao:** Writing – review & editing, Supervision, Methodology, Funding acquisition.

## Declaration of competing interest

The authors declare that they have no known competing financial interests or personal relationships that could have appeared to influence the work reported in this paper.

## Data Availability

Data will be made available on request.
