# Supplementary material for: Hematoma-inspired injectable composite hydrogels incorporating hybrid metal ion microspheres for accelerated bone regeneration
Source: Mater Today Bio. 2025 Sep 25;35:102342. doi: 10.1016/j.mtbio.2025.102342 (PMC12547220; doi:10.1016/j.mtbio.2025.102342)
Supplement: Multimedia component 1 [file mmc1.docx]

**Supporting Information**

**Hematoma-inspired injectable composite hydrogels incorporating hybrid metal ion microspheres for accelerated bone regeneration**

Fan Cao^a,1^, Jin-Yong Wu^b,1^, Qing-Ning Wang^c,^ Jun-Jie Xiao^b^, Zhu Chen^b^, Ya-Wen Wang,^b^ Zhi-Guo Wang^d,*^, Jia-Zhuang Xu^b^, Zhong-Ming Li^e^, Bai-Song Zhao^f,^^*^

*^a^ Department of cardiovascular surgery, Guangdong Cardiovascular Institute, Guangdong Provincial People's Hospital, Guangdong Academy of Medical Sciences, Southern Medical University, Guangzhou 510080, China.*

*^b^ College of Polymer Science and Engineering, National Key Laboratory of Advanced Polymer Materials, Sichuan University, Chengdu 610065, China*

*^c^ Department of Anesthesiology, The Affiliated Panyu Central Hospital of Guangzhou Medical University, Guangzhou, 511400, China.*

*^d^ Medicine and Engineering Interdisciplinary Research Laboratory of Nursing & Materials, West China Hospital, Sichuan University/West China School of Nursing, Sichuan University, Chengdu 610041, China*

*^e^ West China School of Medicine/West China Hospital, Sichuan University， Chengdu 610041, China*

*^f^ Department of Anesthesiology, Zhujiang Hospital, Southern Medical University, Guangzhou, 510280, China.*

*** Corresponding author:**

E-mail: [zgwang@scu.edu.cn](mailto:zgwang@scu.edu.cn) (Z. W.)

E-mail: [zhaobaisong819@smu.edu.cn](mailto:zhaobaisong819@smu.edu.cn) (B. Z.)

- **Experiments***1. The hemolysis test*

The whole blood was collected from SD rats and anticoagulated with sodium heparin (20 U/mL). A 4% (v/v) blood cell suspension was prepared by mixing 0.2 mL of anticoagulated whole blood with 10 mL of physiological saline, followed by gentle shaking to ensure homogeneity. Each hydrogel sample was immersed in 1 mL of physiological saline, after which 1 mL of the 4% blood cell suspension was added. The negative control consisted of 1 mL of physiological saline mixed with 1 mL of the blood cell suspension, while the positive control contained 1 mL of distilled water mixed with 1 mL of the blood cell suspension. All samples were incubated at 37 °C for 1 hour. After incubation, the samples were centrifuged at 1500 rpm for 10 min, and the optical density (OD) of the supernatant was measured at 545 nm. The hemolysis rate was calculated according to the following formula:

Hemolysis rate (%) = (OD_sample_ − OD_negative control_) / (OD_positive control_ − OD_negative control_) × 100%.

*2. The scratch assay*

The cell migration-promoting capability of the composite hydrogel was assessed using an *in vitro* scratch assay with bone marrow mesenchymal stem cells (BMSCs). The cells were seeded into 24-well plates at a density of 5 × 10⁴ cells per well and cultured in DMEM supplemented with 10% FBS and 1% penicillin-streptomycin. A linear scratch wound was then carefully created in the monolayer using a sterile 200 μL pipette tip. Afterward, the wells were washed twice with PBS to remove dislodged cells. Cell migration into the scratched area was documented at 0 h and 24 h using an inverted phase-contrast microscope. Quantitative analysis of the scratch closure was performed using ImageJ software with the MRI Wound Healing Tool plugin.


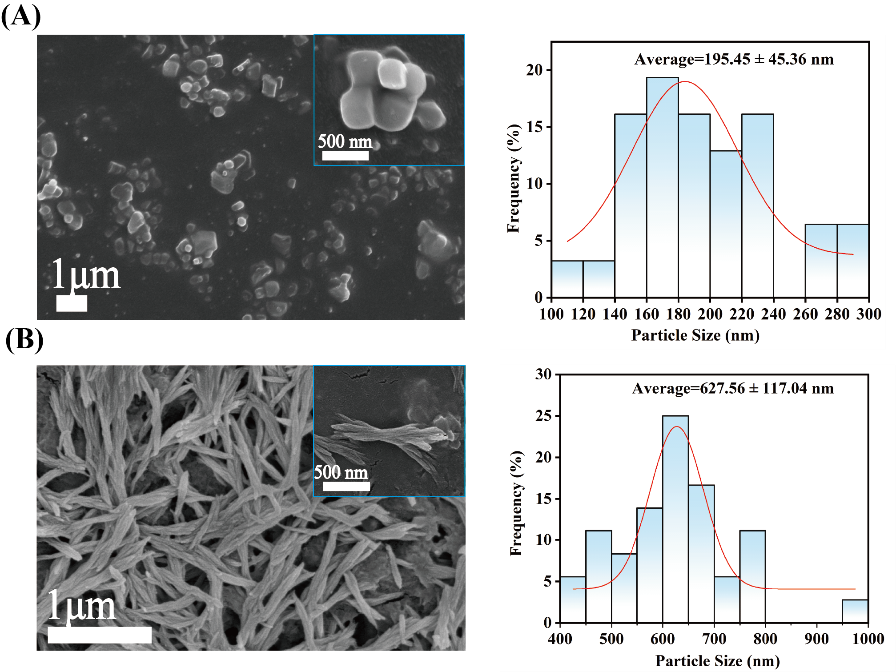


**Fig. S1.** SEM images and the size distribution of MgO and SrO.


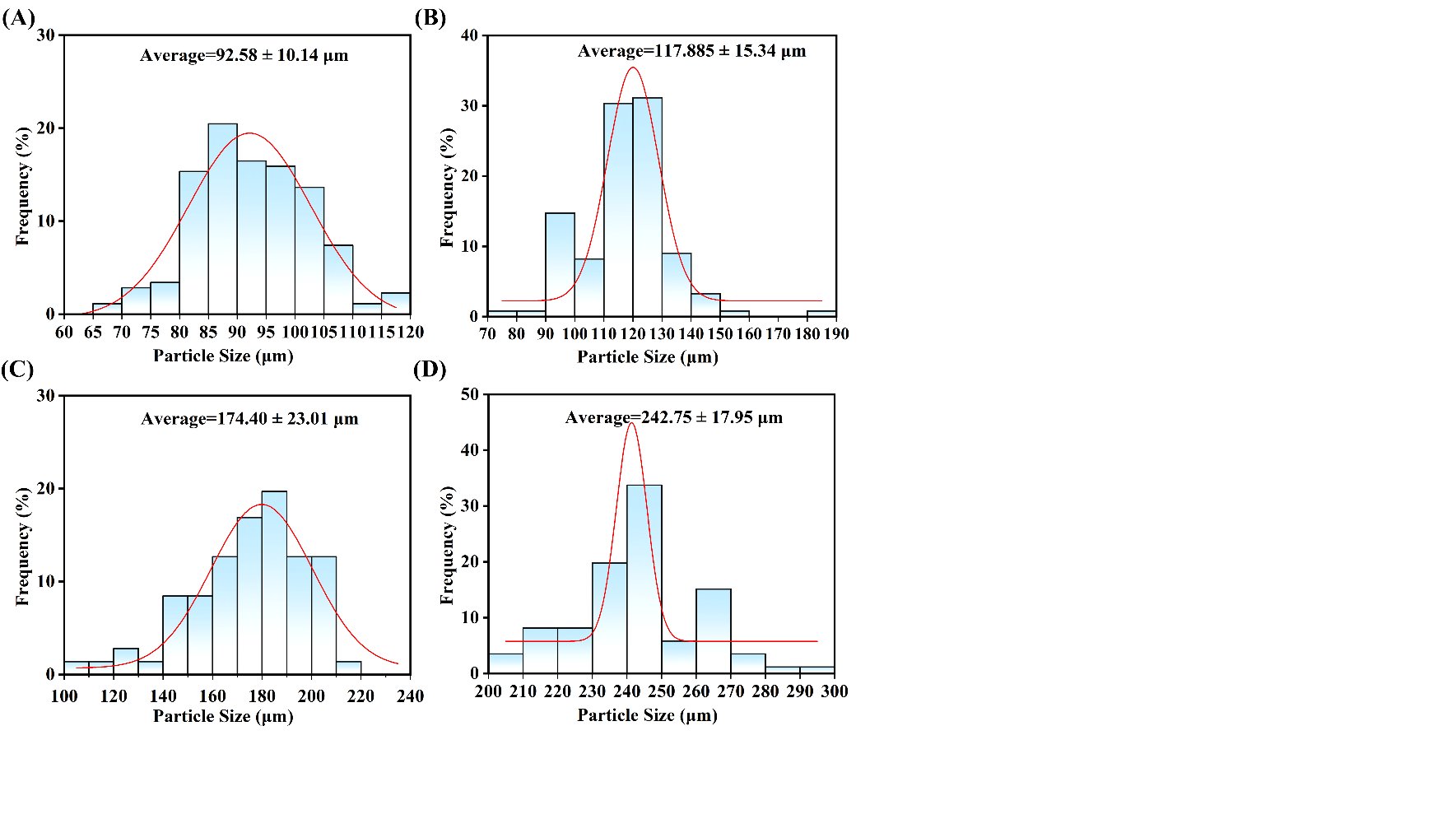


**Fig. S2.** Particle size distributions of (A) PL, (B) Mg@PL, (C) Sr@PL, and (D) MS@PL osteogenic microspheres.


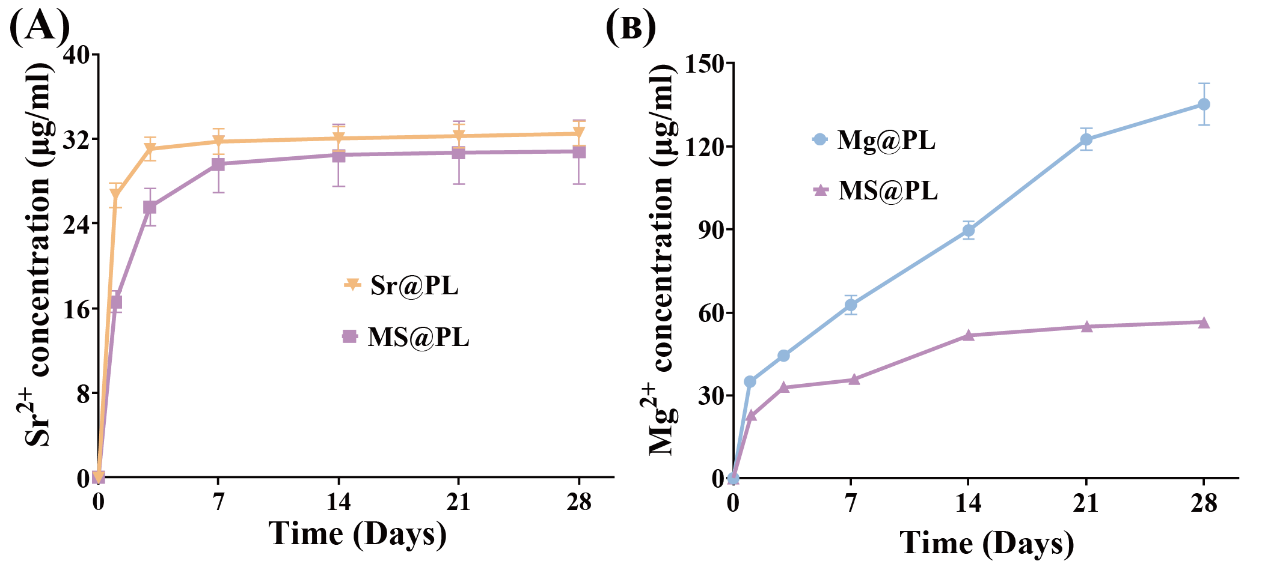


**Fig. S3.** Concentrations of (A) Mg²⁺ and (B) Sr²⁺ released from Mg@PL, Sr@PL, and MS@PL at different release time.

**
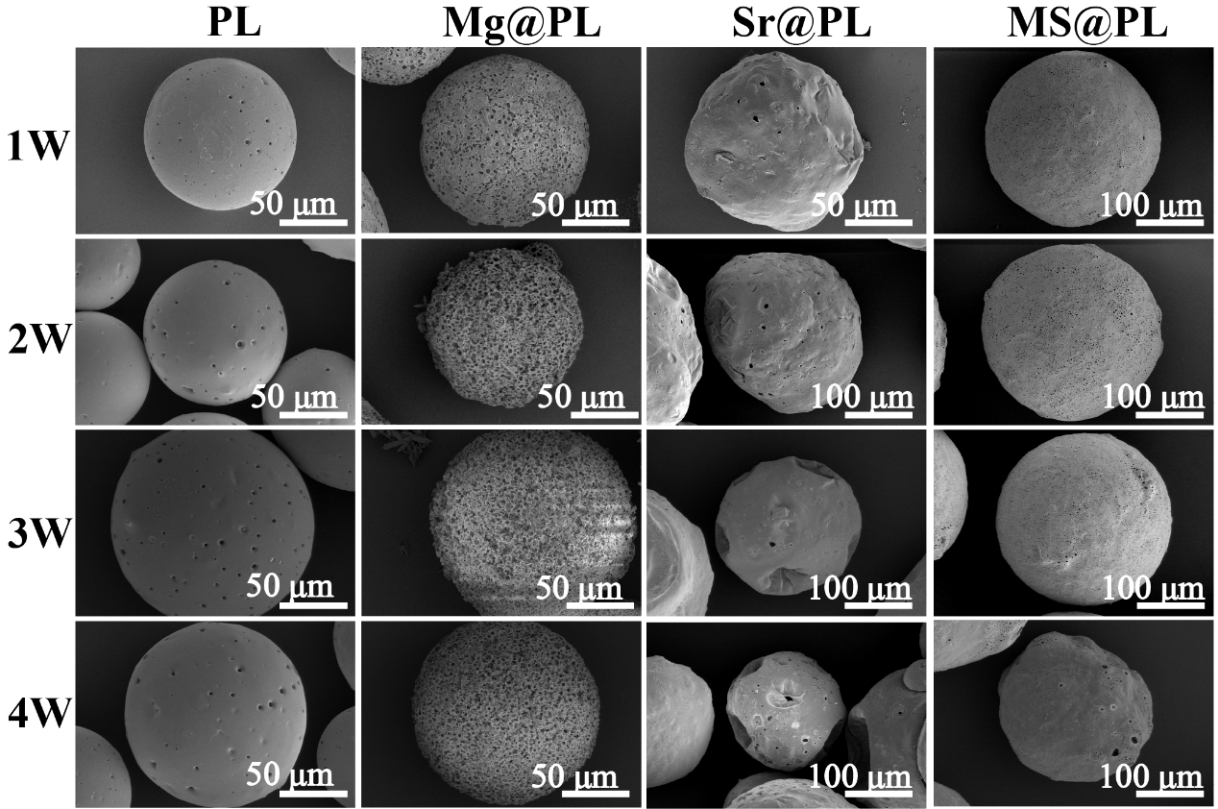
**

**Fig. S4.** Degradation of osteogenic composite microspheres in PBS solution for 28 days.


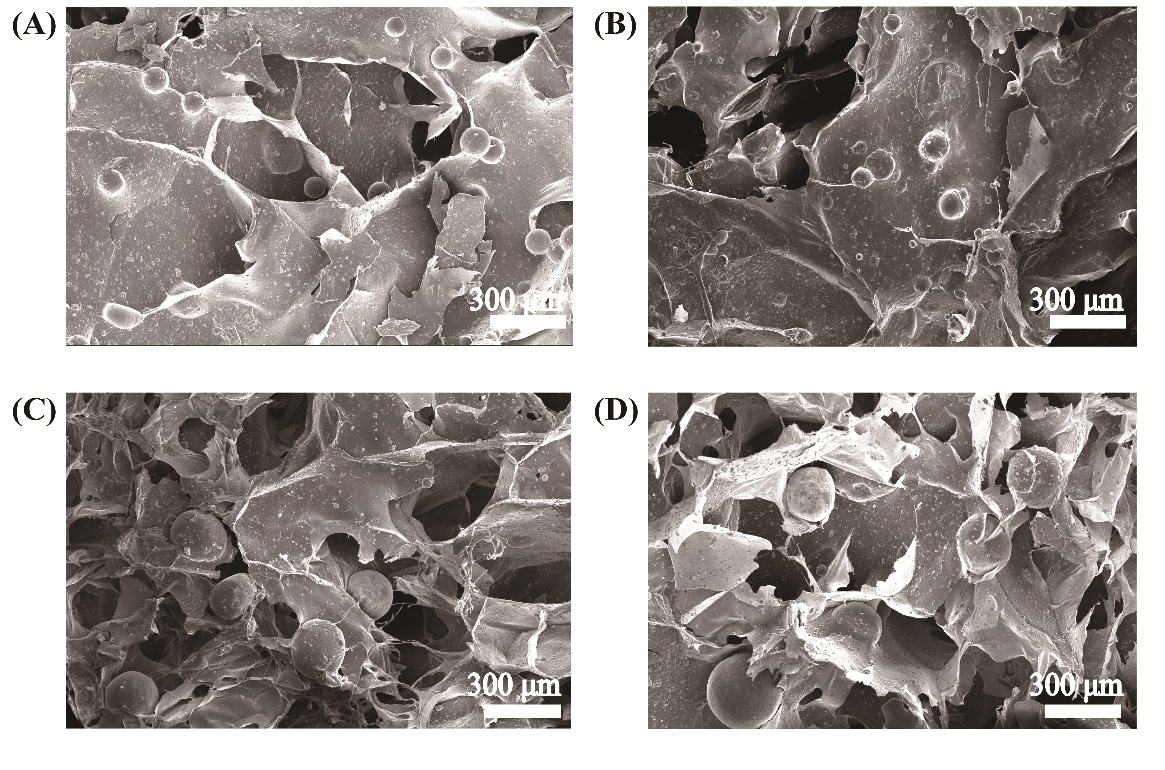


**Fig. S5.** The cross-section SEM images of (A) PS, (B) MPS, (C) SPS and (D) HICP.

**
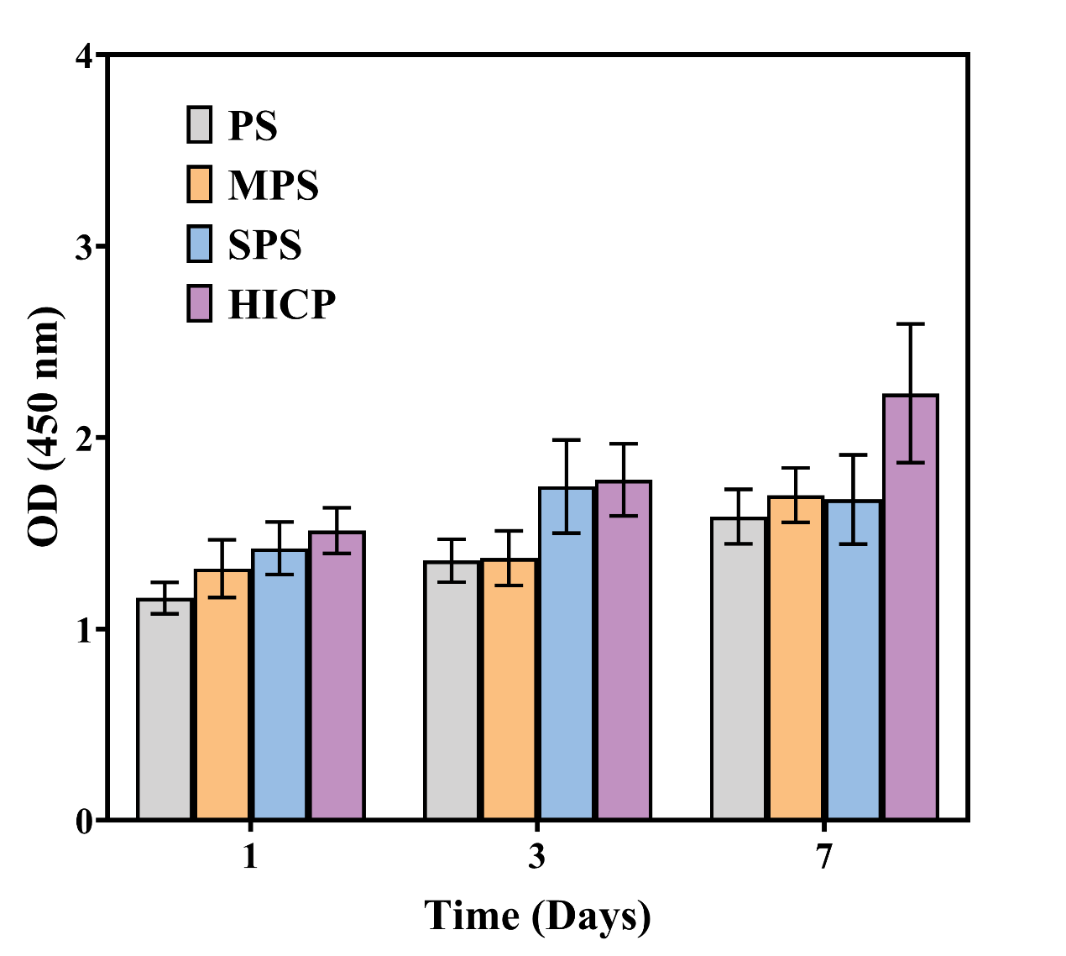
**

**Fig. S6.** Optical density (OD) values at 480 nm of composite hydrogels co-cultured with BMSCs on day 1, 3, and 7.


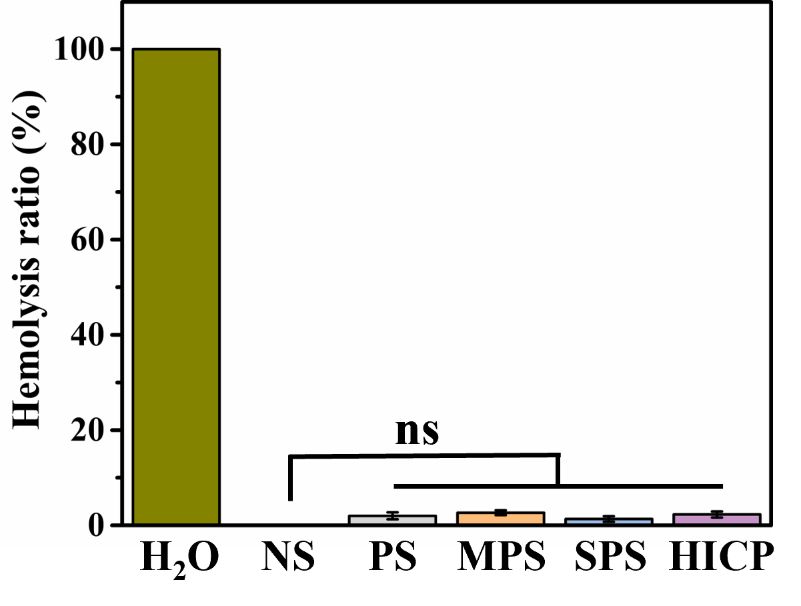


**Fig. S7.** The hemolysis ratio after being treated with different hydrogels.


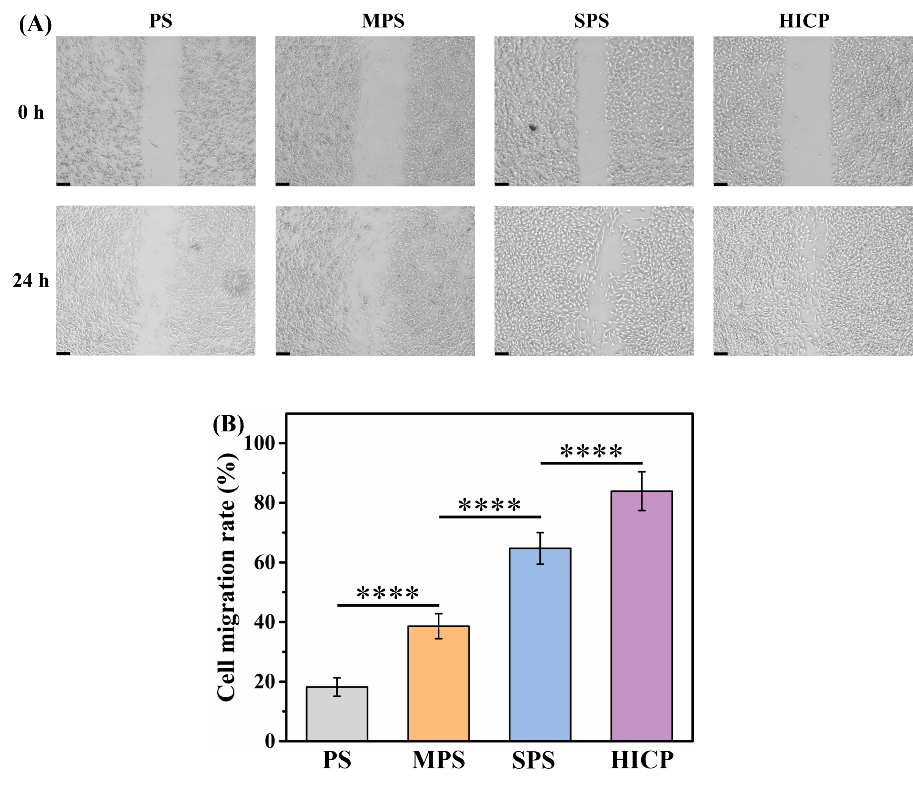


**Fig. S8.** (A) Cell scratch images of BMSCs and (B) the quantitative cell migration rate after contact with the hydrogel extracts for 24 h.

**Table S1.** The ratio of Ca and P for different groups based on the *in vitro* biomineralization experiment.

| **Sample** | **Ca/P ratio** |
| --- | --- |
| HA | 2.15 |
| PL | 2.99 |
| Mg@PL | 2.00 |
| Sr@PL | 2.57 |
| MS@PL | 2.34 |
